# The Circadian Rhythms of STAT3 in the Rat Pineal Gland and Its Involvement in Arylalkylamine-N-Acetyltransferase Regulation

**DOI:** 10.3390/life11101105

**Published:** 2021-10-18

**Authors:** Simona Moravcová, Eva Filipovská, Veronika Spišská, Irena Svobodová, Jiří Novotný, Zdeňka Bendová

**Affiliations:** 1 Department of Physiology, Faculty of Science, Charles University, 128 43 Prague, Czech Republic; simona.moravcova@natur.cuni.cz (S.M.); eva.filipovska@natur.cuni.cz (E.F.); veronika.spisska@natur.cuni.cz (V.S.); jiri.novotny@natur.cuni.cz (J.N.); 2 Department of Sleep Medicine and Chronobiology, National Institute of Mental Health, 250 67 Klecany, Czech Republic; 3 Laboratory of Pain Research, Institute of Physiology, Academy of Sciences of the Czech Republic, 142 20 Prague, Czech Republic; svobodova4irena@gmail.com

**Keywords:** STAT3, arylalkylamine-N-acetyltransferase, pineal gland, circadian rhythms, lipopolysaccharide, rat

## Abstract

In rodents, the melatonin production by the pineal gland is controlled through adrenergic signaling from the suprachiasmatic nuclei and regulation of the principal enzyme in its synthesis, arylalkylamine-N-acetyltransferase (AANAT). In the present study, we identified increased isoprenaline-induced *aa-nat* expression and nocturnal AANAT activity in the pineal glands in response to the silencing of the signal transducer and activator of transcription 3 (STAT3) with siRNA or STAT3 inhibitors WP1066 and AZD1480. This AANAT activity enhancement in vivo did not interfere with light-induced AANAT suppression. Systemic or in vitro lipopolysaccharide (LPS) administration markedly increased *Stat3* expression and STAT3 phosphorylation, but it did not significantly affect AANAT expression or activity. Simultaneous LPS administration and *Stat3* silencing enhanced the *aa-nat* transcription and AANAT activity to a similar extent as *Stat3* inhibition without LPS co-administration. Furthermore, we describe the circadian rhythmicity in *Stat3* expression and the phosphorylated form of STAT3 protein in the rat pineal gland. Our data suggest that the higher nocturnal endogenous level of STAT3 in the pineal gland decelerates or hampers the process of NA-induced AANAT activation or affects the AANAT enzyme stability.

## 1. Introduction

The pineal gland is a neuroendocrine organ whose primary function is the nocturnal production of melatonin, a neurohormone with widespread biological impact. The melatonin synthesis occurs within the pinealocytes and involves N-acetylation of serotonin by arylalkylamine-N-acetyltransferase (AANAT), followed by methylation of the 5-hydroxyl group by hydoxyindole-O-methyltransferase [1]. The pineal gland is innervated with the hypothalamo-pineal pathway, which conveys the timing information from the suprachiasmatic nucleus (SCN) [2]. During the night, the SCN causes an increase in activity of the postganglionic sympathetic fibers and release of noradrenaline (NA) into pineal parenchyma, where it binds to β-AR receptors on pinealocytes. Activation of the intracellular cAMP signaling pathway that leads to phosphorylation of the cAMP response element-binding protein (CREB) and to initiation of *aa-nat* transcription [3,4]. This results in a >100-fold increase in *aa-nat* mRNA level after the onset of darkness, followed by a similar magnitude of increases in AANAT protein level and enzyme activity [5].

Under constant darkness, there are persistent circadian variations in AANAT activity and melatonin levels, with high levels during the night that are rapidly reduced by the exposure to light at night [6,7,8]. In rodents, this is caused by a rapid decrease in cAMP levels, leading to the cessation of transcription and dissociation of the AANAT from activating 14-3-3 protein, followed by AANAT protein degradation [9]. AANAT is considered the key enzyme in melatonin production because most regulatory mechanisms converge at the control of AANAT enzyme activity, and thus, all the changes in AANAT expression and function may be of crucial importance for melatonin-dependent physiology [10,11,12].

The STAT3 belongs to the family of seven proteins that function as transcription factors mediating the effect of cytokines, growth factors, opioids, and some hormones on gene expression of a variety of target genes. Ligand binding to cell-surface receptors activates tyrosine kinases of the Janus (JAK1 and JAK2) or Src family that phosphorylate STAT3 proteins on its tyrosine 705 residue (pSTAT3(y)). Tyrosine-phosphorylated STAT3 make homo- and heterodimers, translocate to the nucleus, and bind to GAS family members of promoter enhancers [13]. The *Stat3* gene promoters also contain GAS elements [14], and thus its expression can be regulated by the positive autoregulatory loop. Serine/threonine kinases have been shown to phosphorylate STAT3 on S727 (pSTAT3(s)), which may recruit transcriptional cofactors, such as p300/CBP, and modulate transcriptional activity of pSTAT3(y) [15,16]. 

The STAT3 is intensively studied because of its involvement in inflammation, cancer, and neurodegeneration [17]. STAT3, however, plays an important role in a wide range of physiological functions, including neurogenesis, energy homeostasis, hormonal regulation, and synaptic plasticity [13]. Despite STAT3 expression being well described in various brain systems [18,19,20,21,22], there is little evidence of its expression in the pineal gland and none is placed in context with its function. Our previous work described the circadian rhythms in Stat3 gene expression and endogenous and LPS-induced STAT3 protein phosphorylation in the SCN. [18,19]. The aim of this work was to describe the rhythmicity of STAT3 in the pineal gland and evaluate its possible role in the regulation of AANAT transcription and activity.

## 2. Materials and Methods

### 2.1. Animal Experiments

Wistar rats (Velaz, Ltd; Prague, Czech Republic) were maintained under a 12/12 h light–dark regime at a temperature of 23 ± 2 °C with free access to food and water. This study was carried out in strict accordance with the recommendations in the Guide for the Care and Use of Laboratory Animals of the National Institutes of Health. The protocol was approved by the Animal Protection Law of the Czech Republic (protocol number: MSMT-31592/2019-4, 2 December 2019). 

#### 2.1.1. The Circadian Rhythmicity of Stat3 mRNA

Adult male rats were maintained under a 12/12 h light–dark regime for 2 weeks before the experiments. On the day of experiment, the animals were released into constant darkness (DD) at the time of dark-to-light transition (designated as circadian time 0; CT0), and they were sacrificed within the first cycle in the darkness in 3-h intervals throughout the 24-h cycle (n = 5). The pineal glands were frozen and used for *Stat3* detection by qPCR. 

#### 2.1.2. The Circadian Rhythmicity of pSTAT3 Levels in Pineal Glands 

Adult male rats were maintained as described above. On the day of experiment, the animals were released into DD and sacrificed in 3-h intervals throughout the 24 h cycle (n = 4). Animals were anesthetized by thiopental (50 mg/kg) and perfused through the ascending aorta with heparinized saline, followed by PBS (0.01 M sodium phosphate/0.15 M NaCl, pH 7.4), and then with 4% paraformaldehyde in PBS. Pineal glands were removed, postfixed for 12 h at 4 °C, cryoprotected in 20% sucrose in PBS overnight at 4 °C, frozen on dry ice, and stored at −80° C until processing by immunohistochemistry.

#### 2.1.3. The Effect of STAT3 Inhibitors on the Day/Night AANAT Activity and Light-Induced Suppression of AANAT Activity

Forty-eight adult male rats were maintained as described above. On the day of experiment, the animals were released into DD and separated into nine groups; three groups received intraperitoneal injection of cell-permeable STAT3 inhibitor WP1066 (A11795, AdooQ Bioscience; 40 mg/kg [23], or selective Janus kinases inhibitor shown to inhibit endogenous, as well as cytokine-induced, STAT3 activation AZD1480 (A10110, AdooQ Bioscience; 40 mg/kg [24], or saline. The treatment was completed during the subjective day at CT4, and animals were sacrificed 5 h later at CT9. Three groups were treated with WP1066, AZD1480, or saline at CT15 and sacrificed at CT20. Three groups were treated with inhibitors and saline at CT15 as described, but 5 h after the treatment with inhibitors, the animals underwent a 1-min light pulse of intensity 30 lux and were sacrificed 20 min after. The pineal glands were frozen and processed by AA-NAT enzymatic activity assay.

#### 2.1.4. The Effect of LPS on the Stat3 Expression

Adult male rats were housed as described above. On the day of experiment, they were separated in two groups and released into DD; animals in one group received an intraperitoneal injection of LPS (1 mg/kg) during the subjective day at CT8, and pineal glands were sampled 4 h later and then in 3-h intervals throughout the 24-h cycle (n = 4). The second group received saline and served as a control (n = 4). The pineal glands were frozen and used for Stat3 detection by qPCR.

#### 2.1.5. The Effect of LPS on STAT3 Phosphorylation

Adult male rats were housed as described above. On the day of experiment, they were separated in two groups and released into DD. Animals in one group received an intraperitoneal injection of LPS (1 mg/kg) during the subjective day at CT6 and were sampled 2 h, 4 h, 6 h, 8 h, 11 h, and 15 h later. The second group received LPS at CT15 and pineal glands were sampled 2 h, 5 h, 8 h, and 24 h later. The animals were anesthetized and perfused through the aortas as described in 2.3, and the pineal glands were stored at −80 °C until processing by immunohistochemistry (n = 4).

#### 2.1.6. The Effect of LPS and WP1066 on AANAT Activity in Pineal Glands

Adult male rats were housed as described above. On the day of experiment, they were separated into three groups (n = 4) and released into DD. Animals in one group received an intraperitoneal injection of LPS (1 mg/kg) during the subjective night at CT15. The second group received ip. injection of STAT3 inhibitor WP1066 (40 mg/kg) and 1 h later the LPS (1 mg/kg). The third group received saline. All animals were sampled at CT20. The pineal glands were frozen and processed by AA-NAT enzymatic activity assay.

### 2.2. Organotypic Cultures

Organotypic cultures were prepared from 16-day-old rats. Pineal glands were removed after rapid decapitation and placed onto cell culture inserts with a pore size of 0.001 mm (BD Falcon, Tewksbury, MA, USA) in 6-well plates (Falcon). Inserts were submerged in 1 mL of Neurobasal A medium supplemented with 2% serum-free B-27, 50 U/mL penicillin, 50 µg/mL streptomycin, and 0.5 mM L-glutamine (Thermo Fisher Scientific, Waltham, MA); saturated with 95% air and 5% CO_2_ mixture; and incubated in a humidified 5% CO_2_ atmosphere at 37 °C. Organotypic cultures were allowed to stabilize, and after 3 days of cultivation, they were stimulated either with WP1066 inhibitor (WP, 5 µM) for 6 h, LPS (10 µg/mL of media) for 5 h, isoprenaline (ISO, Sigma-Aldrich (St. Louis, MO, USA), I6504; 2 µM) for 4 h, or their mutual combinations. Organotypic cultures were then frozen and processed by AA-NAT enzymatic activity assay.

### 2.3. Primary Cultures

Postnatal day 5 newborn rats were euthanized by decapitation. Pinealocytes were prepared by trypsinization of pineal glands followed by mechanical dispersion according to the established method and used for the isolation of primary cultures from suprachiasmatic nucleus [25,26]. Cells from 16 animals (∼150,000 cells) were placed on 24 coverslips coated with a 1% poly-L-lysine solution (Sigma) and cultivated in 24-well plates in Neurobasal A medium supplemented with 2% serum-free B-27 and 0.5 mM L-glutamine in a humidified 5% CO_2_ atmosphere at 37 °C. After 3 days of cultivation, pinealocytes were transfected using lipofection with Stat3 siRNA. The successful transfection and subsequent decline in STAT3 expression levels were proved by sodium dodecyl sulphate-polyacrylamide gel electrophoresis (SDS-PAGE). After 2 days of incubation with siRNA, cells were treated with LPS (10 µg/mL media) for 5 h, ISO (2 µM) for 4 h, or their mutual combination. Cells were collected into RNAzol RT (Molecular Research Center) and stored at -80 °C until use for isolation of RNA. 

### 2.4. Isolation of RNA and Quantitative Real-Time PCR

Total RNA was isolated from primary cultures using RNAzol® RT (Molecular Research Center) and was extracted with Direct-zol RNA MiniPrep (Zymo research, Irvine, CA, USA) according to the manufacturer´s instructions. One µg of total RNA was converted to cDNA using a High-Capacity cDNA Reverse Transcription kit (Applied Biosystems, Waltham, MA, USA). Samples of cDNA (1 µL) were amplified in 20 µL of a PCR reaction mixture containing 5x HOT FIREPol® Probe qPCR Mix Plus (Baria, Prague, Czech Republic) plus TaqMan assay (Applied Biosystems). The expression levels of *aa-nat*, *Icer*, and *Stat3* were measured by real-time RT-PCR using pre-developed TaqMan® assays (*aa-nat*: Rn01461110_m1, *Icer*: Rn04338541_m1, *Stat3*: Rn00680715_m1, Applied Biosystems). All the qPCR reactions were performed in triplicate in a LightCycler 480 Instrument (Roche Life Science, Indianapolis, IN, United States). The mean of the crossing point (Cp) obtained from the qPCR was normalized to the level of the housekeeping gene HPRT (Rn01527840_m1, Applied Biosystems) and used to analyze relative gene expression using the ΔΔCt method [27].

### 2.5. Immunohistochemistry

Pineal glands were sectioned into series of 20-µm-thick sections. Levels of pSTAT3 (Tyr705; rabbit monoclonal, 9145, AB_561305, Cell Signaling Technology, Denver, MA, USA), and pSTAT3 (Ser727; rabbit polyclonal, 9134, AB_331589, Cell Signaling Technology, Denver, MA, USA) were assessed by immunohistochemistry with the avidin-biotin method with diaminobenzidine as the chromogen (Vectastain ABC kit; Vector Laboratories, Burlingame, CA). At first, sections were treated with 1% NaOH and 1% H_2_O_2_ for 20 min, 0.3% glycine for 10 min, and 0.03% sodium dodecyl sulphate for 10 min, as described by [28]. After being rinsed in PBS, sections were blocked with 2% normal goat serum and incubated overnight in primary antibody (1:300). Immunopositive cells were manually tagged in an approximate 0.5 mm2 area and counted in Image J (NIH) by two researchers, who were blind to the experimental groups, with similar results. The values for each animal were calculated as the mean number of the 3–4 sections from one pineal gland.

### 2.6. AA-NAT Enzymatic Activity Assay

AA-NAT activity was determined as described previously [29]. Briefly, pineal glands were stored frozen in dry ice until homogenization in a reaction mixture of 0.1 M phosphate buffer (pH 6.8) containing 0.25 mmol [1-14C] acetyl coenzyme A (specific activity, 37 Mbq/mmol) and 10 mmol tryptamine hydrochloride in a final volume of 100 μL. The reaction mixture was incubated at 37 °C for 20 min. At the end of the incubation period, the reaction was stopped by the addition of 1 mL of chloroform. After 1 min of vortexing, the aqueous phase was removed, and the organic phase was washed three times with 0.2 mL of phosphate buffer (pH 6.8). The organic phase was transferred to a scintillation vial and dried by evaporation. Radioactive acetylated product was determined by scintillation counting. AA-NAT activity was expressed as nanomoles of N-acetyltryptamine formed per h per 1 mg of pineal tissue.

### 2.7. Western Blotting

Immunoblotting has been done as described in Bendová and Sumová [30]. Pineal glands maintained in an ice bath were sonicated two times for 5 s in cold TMES buffer (20 mM Tris, 3 mM MgCl2, 1 mM EDTA, 0.25 M sucrose; pH 7.4) containing protease and phosphatase inhibitors (Complete Protease Inhibitor Cocktail and PhosSTOP, Roche Diagnostics, Basel, Switzerland). Samples of pineal gland protein extracts were measured by BCA assay and diluted in a Laemmli sample buffer to equal amounts of protein. The 20 µg of total protein were separated on standard 10% polyacrylamide gels and electroblotted onto a nitrocellulose membrane (Protran BA85, GE Healthcare, Little Chalfont, Buckinghamshire, UK) and then incubated overnight at 4 °C with specific primary antibodies (Stat3: rabbit monoclonal, 4904, AB_10693927, Cell Signaling Technology, Denver, MA, USA, dilution 1:2000; pStat3(y): rabbit monoclonal, 9145, AB_561305, Cell Signaling Technology, Denver, MA, USA, dilution 1:2000; pStat3(s): rabbit polyclonal, 9134, AB_331589, Cell Signaling Technology, Denver, MA, USA, dilution 1:2000); TLR4: mouse monoclonal, sc-293072, Santa Cruz Biotechnology, dilution 1:2000). Then, membranes were washed and processed with an appropriate horseradish peroxidase-conjugated secondary antibody, and immunoreactive bands were visualized using SuperSignal West Dura Substrate (Pierce Biotechnology, Rockford, IL, USA) according to manufacturer’s instructions. The immunoblots were scanned (EPSON Perfection V600 Photo).

### 2.8. Data Analysis and Statistical Procedures

The rhythmicity of the *Stat3* gene expression and pSTAT3(y) was evaluated using a one-way ANOVA to determine the effect of time and was further analyzed using cosinor analysis defined by the following equation, with a constant wavelength of 24 h: [Y = mesor + (amplitude ∗ −cos(2 ∗ *p* ∗ (X − acrophase)/wavelength]. A horizontal line-fit model (H0) and cosinor analysis were used as the alternative hypothesis. The rhythmicity was approved when both tests were significant. Unpaired two-tailed *t* tests were used for single column comparison, and multiple *t*-tests with the Sidak–Bonferroni post-hoc test were used for the effect of in vivo LPS administration. 

## 3. Results

### 3.1. Circadian Rhythmicity of Stat3 Expression and the Level of pSTAT3(y) in the Pineal Gland

**Figure 1:**
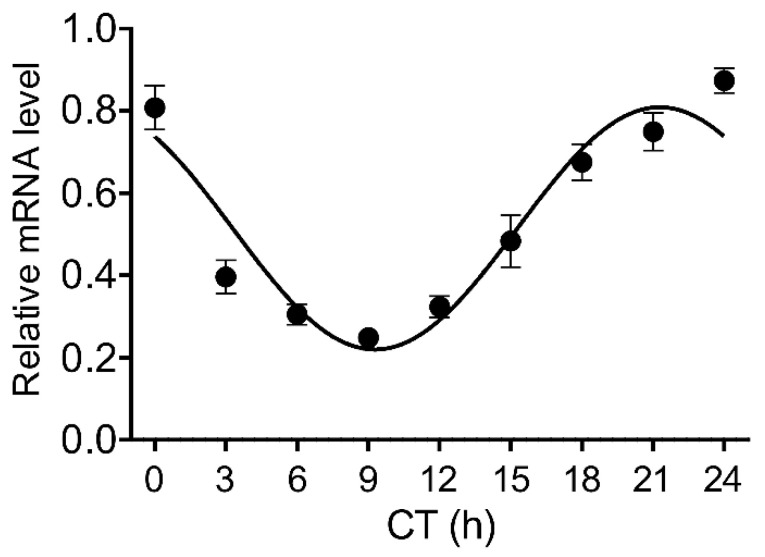
The circadian rhythmicity of Stat3 mRNA expression in adult rat pineal glands determined by qPCR. The normalized values are expressed as relative mRNA levels. The data indicated by dots were fitted with a cosine curve using the cosinor analysis method. Each point represents the mean ± SEM from five animals.

To determine whether the Stat3 shows circadian rhythmicity in the rat pineal gland, we performed qPCR to determine changes in mRNA level and immunohistochemistry to reveal changes in the phosphorylated form of its protein. We found that the Stat3 mRNA expressed the circadian rhythm in the pineal gland (Figure 1; one-way ANOVA: F [8, 36] = 23.92; *p* < 0.0001). The cosinor analysis rejected the null hypothesis (horizontal line) and confirmed the significant circadian rhythmicity for the Stat3 (*p* < 0.0001).

**Figure 2:**
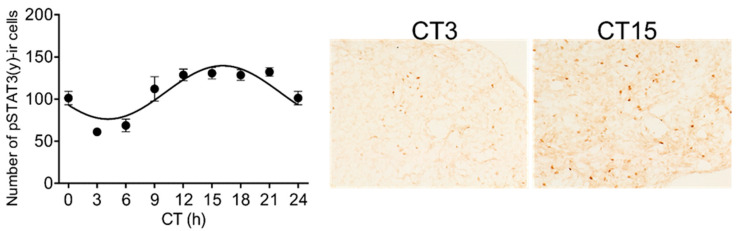
The circadian rhythmicity of pSTAT3(y) level in adult rat pineal glands determined using immunohistochemistry. The data indicated by dots were fitted with a cosine curve using the cosinor analysis method. Levels of immunopositive cells were assessed for approx. 0.5 mm^2^—the area demonstrated in representative autoradiographs on the right (magnification 400x). Each point represents the mean ± SEM from four animals.

The significant rhythmicity was also confirmed for the level of the pSTAT3(y) (Figure 2; one-way ANOVA: F [8, 27] = 15.68; *p* < 0.0001; cosinor analysis: *p* < 0.0001).

### 3.2. The STAT3 Inhibition Enhances ISO-Induced Transcription and Activation of AANAT

**Figure 3:**
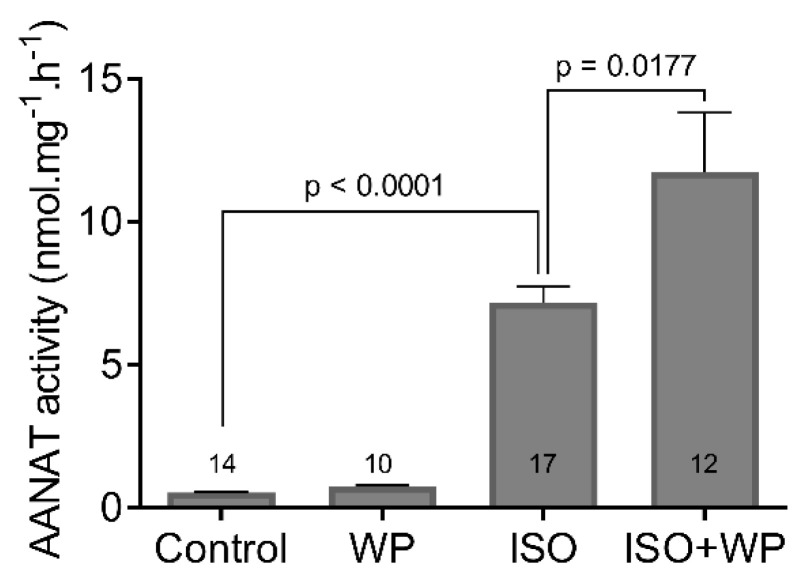
The activity of AANAT in organotypic cultures of pineal glands. The cultures were stimulated with STAT3 inhibitor WP1066 (WP; 5 μM) for 6 h, isoprenaline (ISO; 2 μM) for 4 h, or pretreated with WP1066 for 2 h followed by ISO for 4 h (ISO + WP). The data are the mean of 10–17 pineal glands from four independent experiments (numbers within the columns). The statistical significance is shown with unpaired two-tailed *t* test.

In search of the role of STAT3 in pineal physiology, we focused on its effect on AANAT, the most intensively studied enzyme in the melatonin synthetic pathway. We prepared organotypic cultures of pineal gland and treated them with the cell-permeable inhibitor of STAT3 signaling WP1066 for 4 h, non-selective β-AR agonist isoprenaline (ISO) for 6 h, or their respective combinations (Figure 3). ISO significantly induced AANAT activity (Control vs. ISO: t = 10.81, df = 29, *p* < 0.0001) as expected [31]. Our data showed that, while WP1066 alone had no effect, when combined with ISO, it enhanced the AANAT activity by 63% compared to ISO alone (ISO vs. ISO + WP: t = 2.520, df = 27, *p* = 0.0177).

**Figure 4:**
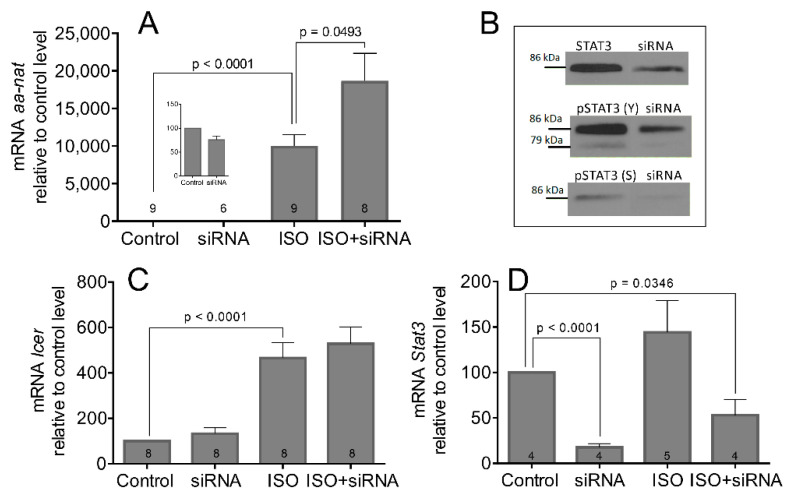
The changes in aa-nat expression (**A**), Icer mRNA (**C**), and Stat3 mRNA (**D**) in the rat pinealocytes. The insert in (**A**) indicates the first two column values in a different scale of the y-axis. (**B**) Representative western blots of STAT3 and its phosphorylated forms after transfection of pinealocytes with Stat3 siRNA (The original western blots are in the Supplementary Materials Figure S1). After 2 days of incubation with Stat3 siRNA, cells were treated with 2 µM ISO for 4 h. Data are presented as the means ± SEM of four to nine experiments (different dissections; numbers within the columns). The statistical significance is shown with unpaired two-tailed *t* test.

As the nocturnal increase in AANAT activity requires a neo-transcription of its gene and a neo-synthesis of its protein [5,9], we tested whether the changes in AANAT activity reflected the changes in aa-nat transcription. We used primary cultures of pinealocytes that were transfected with Stat3 siRNA, and 2 days later, they were stimulated with ISO for 6 h. The level of aa-nat transcript was assayed by qPCR (Figure 4A). The results show that the changes of aa-nat mRNA resembles that of its enzyme activity, and the siRNA alone does not significantly affect the basal level of the aa-nat transcript. Concurrent β-AR stimulation with ISO leads to 86% enhancement of aa-nat transcription compared to ISO alone (Control vs. ISO: t = 7.775, df = 16, *p* < 0.0001; ISO vs. ISO + siRNA: t = 2.187, df = 15, *p* = 0.0493). The effectiveness of siRNA transfection for the level of STAT3 protein and its phosphorylated forms is shown in Figure 4B.

Besides aa-nat, the inducible cAMP early repressor (Icer) is another NA-induced gene that is significant for pineal physiology. It inhibits the cAMP-stimulated genes and may negatively affect aa-nat transcription [32]. Figure 4C shows that the Icer mRNA can be induced by ISO (Control vs. ISO: t = 5.373, df = 14, *p* < 0.0001). However, unlike the aa-nat, the Stat3 siRNA did not upregulate ISO-induced Icer mRNA levels (*p* = 0.2689). Figure 4D shows significant downregulation of Stat3 mRNA level after siRNA transfection (Control vs. siRNA: t = 20.71, df = 6, *p* < 0.0001; Control vs. ISO + siRNA: t = 2.722, df = 6, *p* = 0.0346), suggesting the positive regulatory loop that may exist in the pineal gland between the STAT3 protein and its own gene. The stimulation with ISO did not significantly change the Stat3 expression in this experiment.

### 3.3. The STAT3 Inhibitors Enhances AANAT Activity Only during Subjective Night When the Pineal Gland Receives Adrenergic Stimulation from the SCN but Have No Effect on Light-Induced Downregulation of AANAT Activity

**Figure 5:**
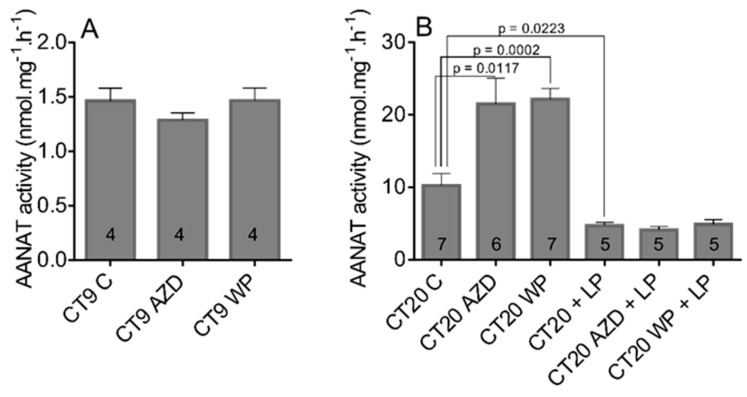
The activity of AANAT in adult rat pineal glands. Animals were treated with STAT3 inhibitors, WP1066 (WP; 40 mg/kg) or AZD1480 (AZD; 40 mg/kg), or a combination of inhibitors with 1 min light pulse (LP). Controls (C) were treated with vehiculum. Animals were injected either during the subjective day at CT4 (**A**) or during the subjective night CT15 (**B**) and euthanized 5 h later. For better clarity, there is a difference in the y-axis scale between (**A**,**B**). The data are the mean of four to seven pineal glands (numbers within the columns). The statistical significance is shown with unpaired two-tailed *t* test.

The results from in vitro experiments suggested that STAT3 does not affect the aa-nat transcription and activity without concurrent stimulation of β-AR signaling. ISO is often used in in vitro conditions to substitute the night-time adrenergic signaling from the SCN. Therefore, to approach the natural conditions, we performed experiments in which the rats were treated with STAT3 inhibitor WP1066 and with AZD1480 to prevent the phosphorylation and activation of STAT3, both during the subjective day (CT4) and subjective night (CT15). The activity of AANAT was measured by radioenzymatic assay in pineal glands collected 5 h later (Figure 5). Our data showed that, while neither inhibitor changed the AANAT activity during the subjective day (Figure 5A), when administered during subjective night at CT15, AZD1480 enhanced the AANAT activity by 110% and WP1066 by 116% compared to the control level (Figure 5B; CT20 C vs. CT20 AZD: t = 3.017, df = 11, *p* = 0.0117; CT20 C vs. CT20 WP: t = 5.302, df = 12, *p* = 0.0002). 

In parallel with this experiment, we tested a hypothesis that STAT3 inhibitors, due to their enhancing effect, may interfere with the light-induced AANAT suppression. The 1-min light pulse of intensity 30 lux significantly decreased the AANAT activity, but administration of either inhibitor had no effect (Figure 5B; CT20 C vs. CT20+LP: t = 2.699, df = 10, *p* = 0.0223; CT20 C vs. CT20 AZD+LP: t = 2.991, df = 10, *p* = 0.0136; CT20 C vs. CT20 WP+LP: t = 2.569, df = 10, *p* = 0.0280).

### 3.4. Bacterial Endotoxin Lipopolysaccharide Induces STAT3 Expression and Phosphorylation

**Figure 6:**
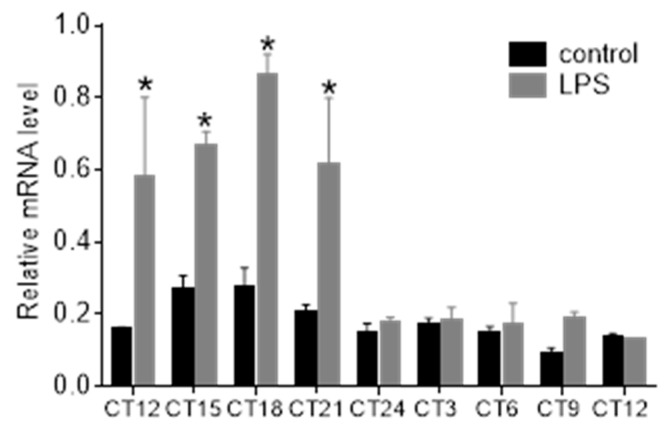
LPS-induced changes in *Stat3* gene expression in rat pineal glands. Adult rats were injected with LPS (1 mg/kg) during the subjective day at CT8, pineal glands were sampled 4 h later and then in 3-h intervals throughout the 24-h cycle. The normalized values are expressed as relative mRNA levels. * indicates the *p* < 0.001 of multiple *t*-tests with the Sidak–Bonferroni post hoc test. Each point represents the mean ± SEM from four animals.

**Figure 7:**
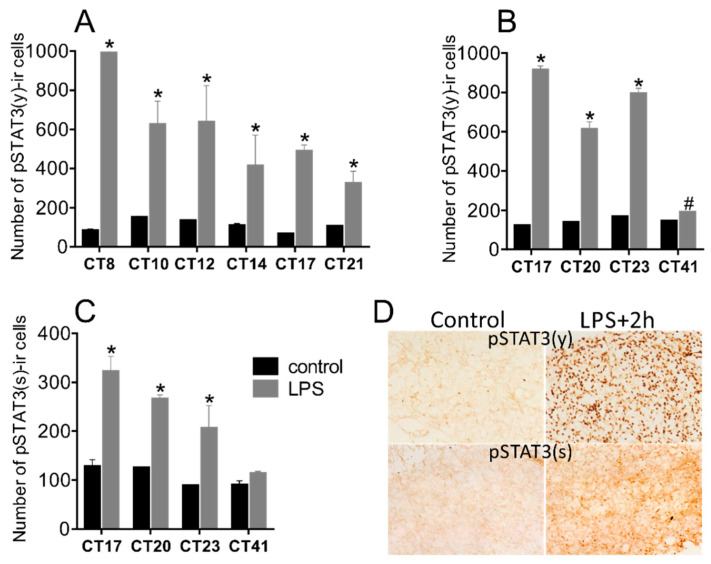
Effect of acute systemic LPS administration on pSTAT(y) (**A**,**B**) and pSTAT(s) (**C**) in rat pineal glands. Adult rats were injected with LPS (1 mg/kg) either during the day at CT6 and sampled 2 h, 4 h, 6 h, 8 h, 11 h, and 15 h later (**A**), or at night at CT15 and sampled 2 h, 5 h, 8 h, and 24 h later (**B**,**C**). Levels of immunopositive cells were assessed for approx. 0.5 mm2—the area demonstrated in representative autoradiographs (**D**, magnification 400x). Each point represents the mean ± SEM from four animals. * indicates the *p* < 0.001 and # indicates *p* < 0.01 of multiple *t*-tests with the Sidak–Bonferroni post hoc test.

In our previous study, we revealed that systemic LPS administration rapidly induced expression and phosphorylation of STAT3 on tyr705 (pSTAT(y)) and ser727 (pSTAT3(s)) in the SCN [18]. Here, we show that LPS upregulates *Stat3* mRNA (Figure 6) and phosphorylated levels of the STAT3 protein (Figure 7) in the pineal gland. Multiple *t*-tests with the Sidak–Bonferroni method showed that the mRNA level increased significantly after administration of LPS at CT8 4 h later, at CT12 (*p* = 0.0003), CT15 (*p* = 0.0002), CT18 (*p* < 0.0001), and CT21 (*p* = 0.0001). At CT24 (i.e., 16 h after the injection), the *Stat3* mRNA level was comparable with the control group. Similarly, as in Figure 1, the *Stat3* expression of control animals showed circadian rhythmicity (one-way ANOVA: F [8, 24] = 3.709; *p* = 0.0055; cosinor analysis: *p* = 0.001; Figure 6).

The acute systemic LPS administration (1 mg/kg) at both, during the early night, or in the middle of the subjective day induced strong STAT3 phosphorylation on tyrosine (Figure 7A,B), as well as on serine (Figure 7C). The effect lasted for several hours; the last measured significant enhancement compared to controls was 15 h after the daytime injection. One day after the night-time injection, indicated at CT41, the level of pSTAT(y) was still significantly elevated.

### 3.5. The LPS and LPS-Induced STAT3 Does Not Affect the Transcription and Activation of AANAT

**Figure 8:**
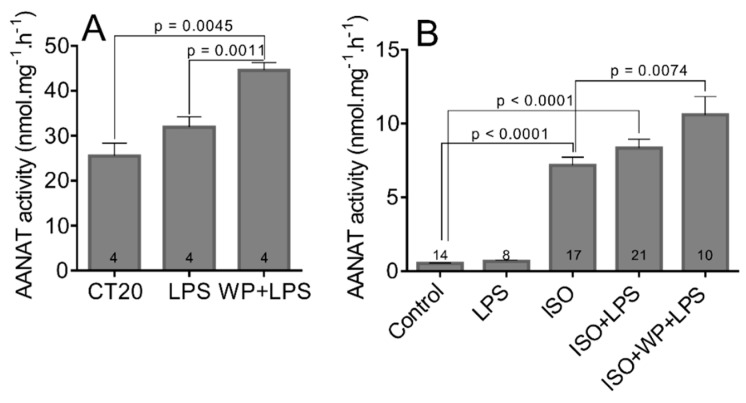
The activity of AANAT in adult rat pineal glands (**A**) and in organotypic cultures of pineal glands (**B**). At CT15, four adult males were treated with LPS (1 mg/kg) or with STAT3 inhibitors WP1066 (40 mg/kg) for 1 h followed by the LPS for 4 h. The pineal glands were sampled 5 h later. The organotypic cultures were stimulated with LPS (10 µg/mL) for 5 h, isoprenaline (ISO; 2 μM) for 4 h, a combination of both (ISO + LPS), or were pre-treated with STAT3 inhibitor WP1066 (WP; 5 μM) for 1 h. The data are the mean of 8 to 21 pineal glands from four independent experiments (numbers within the columns). The statistical significance is shown with unpaired two-tailed *t* test.

Next, we asked whether the LPS-induced STAT3 could be involved in the regulation of AANAT activity. We injected the rats with LPS or with LPS combined with WP1066 at CT15 and harvested their pineal glands 5 h later (Figure 8A,B). Our results showed that the AANAT activity was not significantly changed by the LPS in both experiments, but AANAT activity increased by 75% when animals were co-treated with a STAT3 inhibitor (CT20 vs. WP+LPS: t = 4.693, df = 6, *p* = 0.0045; CT20 vs. LPS: t = 4.408, df = 6, *p* = 0.0011).

To isolate the pineal glands from the context of blood circulation and sympathetic innervation, we prepared organotypic cultures and treated them with LPS, WP1066, and ISO or their respective combinations (Figure 8B). Our data show that, while LPS and WP1066 (for WP1066 see Figure 3) alone did not change the AANAT activity, when combined with ISO, they caused an increase in AANAT activity. The combination of ISO, LPS, and WP1066 showed 50% upregulation compared to ISO alone (Control vs. ISO: t = 10.81, df = 29, *p* < 0.0001; Control vs. ISO+LPS: t = 10.62, df = 33, *p* < 0.0001; ISO vs. ISO+WP+LPS: t = 2.905, df = 25, *p* = 0.0074).

**Figure 9:**
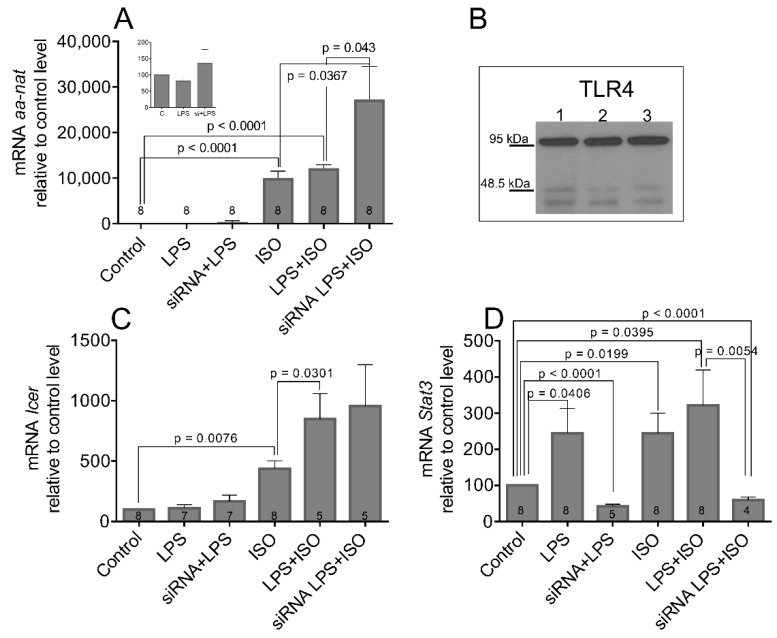
The changes in *aa-nat* expression (**A**), *Icer* mRNA (**C**), and *Stat3* mRNA (**D**) in the rat pinealocytes. The insert in (**A**) indicates the values of the first three columns with a difference in the y-axis scale. (**B**) Representative western blots of TLR4 receptor in three randomly selected pinealocytes cultures. The original western blots are in the Supplementary Materials Figure S2. After 2 days of incubation with *Stat3* siRNA, cells were treated with LPS (10 µg/mL) for 5 h, ISO (2 µM) for 4 h, or with LPS for 1 h followed by ISO for 4 h. Data are presented as the means ± SEM of four to eight experiments (different dissections; numbers within the columns). The statistical significance is shown with unpaired two-tailed *t* test.

Similar results have been obtained for *aa-nat* expression using cultured pinealocytes (Figure 9A). The treatment with LPS alone did not change the *aa-nat* expression and did not significantly upregulate ISO-induced transcription. Only co-transfection with *Stat3* siRNA enhanced ISO-induced *aa-nat* transcription by 170% (Control vs. ISO: t = 8.230, df = 14, *p* < 0.0001; Control vs. ISO+LPS: t = 18.12, df = 14, *p* < 0.0001; ISO vs. siRNA+LPS+ISO: t = 2.350, df = 14, *p* = 0.0367; LPS+ISO vs. siRNA+LPS+ISO: t = 2.242, df = 14, *p* = 0.043). 

TLR4 is a membrane-bound receptor that is well known for recognizing LPS. The molecular weight of TLR4 is approximately 95 kDa. Although the presence of a TLR4 receptor sensitive to LPS has been already found in the pineal gland [33], we checked its presence in three randomly chosen primary culture preparations (Figure 9B) with similar results. 

In contrast to *aa-nat*, expression of *Icer* was upregulated by concurrent stimulation with ISO and LPS (Control vs. ISO: t = 3.257, df = 14, *p* = 0.0076; ISO vs. LPS+ISO: t = 2.488, df = 11, *p* = 0.0301), but similarly to a previous experiment, *Stat3* siRNA had no additive effect. *Stat3* mRNA has been significantly reduced in primary cultures with siRNA, regardless of co-stimulants (Control vs. siRNA+LPS: t = 11.49, df = 11, *p* < 0.0001; Control vs. siRNA+LPS+ISO: t = 7.595, df = 10, *p* < 0.0001; LPS+ISO vs. siRNA+LPS+ISO: t = 1.850, df = 10, *p* = 0.0054). LPS induced *Stat3* mRNA in cultures, similar to in vivo (Control vs. LPS: t = 2.274, df = 14, *p* = 0.0406). Administration of ISO enhanced expression of *Stat3* significantly (Control vs. ISO: t = 2.627, df = 14, *p* = 0.0199), and co-stimulation with LPS had small additive power (Control vs. LPS+ISO: t = 2.270, df = 14, *p* = 0.0395).

## 4. Discussion

The present study, similar to our previous study [20], demonstrates that *Stat3* transcription in the rat pineal gland expresses circadian rhythm with maximum during the subjective night. Furthermore, here we show that also the level of pSTAT3(y) demonstrates circadian rhythmicity. Similarly, the daily rhythm in the *Stat3* expression and STAT3 phosphorylation has been shown in the SCN [18,19,20], but not in the retina [20], which suggests that the STAT3 rhythmicity is not strictly a property of circadian pacemakers but may have more complex tissue-specific regulation. Common with *aa-nat*, the *Stat3* gene contains a CREB binding site in its promoter [34,35]. Although CREB regulates hundreds of genes in many tissues, its action in the pineal gland and SCN is clock-controlled [11,36] and may provide the pathway for circadian regulation of *Stat3* expression in these tissues.

The majority of reports concerning the STAT3 focus on the regulation of genes involved in cancer and inflammation, and its transcriptional activity is mostly studied in the context of its aberrant upregulation and activation [37]. However, in the pineal gland, we show the circadian rhythmicity in basal *Stat3* expression and phosphorylation. As the pineal gland’s most fundamental function is melatonin production, we asked whether this endogenous STAT3 could be involved in the regulation of activity and transcription of the rate-limiting enzyme of melatonin synthesis, AANAT. This enzyme is considered to be a major regulatory node on which multiple signaling pathways affecting the melatonin level converge [10,11,12] and could, thus, respond to manipulation of the STAT3 level or activity.

We used three systems, organotypic cultures, primary cultures, and in vivo systemic treatment with STAT3 inhibitors. The STAT3 silencing in primary cultures with siRNA and the treatment of organ cultures with the STAT3 inhibitor WP1066 resulted in more than a 60% increase in ISO-induced AANAT activity and more than an 80% upregulation of ISO-induced *aa-nat* transcription. The β-AR signaling in the rodent pineal gland is activated via a multisynaptic pathway from the SCN during the night, and ISO is used in in vitro conditions to substitute this night-time derived adrenergic signaling [31]. As the STAT3 inhibition repeatedly showed the effect only with concurrent stimulation with ISO, we assumed that, in natural conditions, the STAT3 action might be restricted to the night-time. Therefore, we performed in vivo experiments, in which we injected the animals during the subjective day and subjective night with inhibitor WP1066 and for control with inhibitor AZD1480. The results clearly supported our hypothesis, and more than 110% enhancement of nocturnal, but not daytime, AANAT activity in the pineal gland by both inhibitors proved that STAT3 affects the AANAT only during night hours. Thus, our results suggest that the higher nocturnal endogenous STAT3 level in the pineal gland may restrict the AANAT function switched on via SCN - β-AR pathway. 

There are many mechanisms that modulate the transcription and activity of AANAT, which may also interact with STAT3 endogenous activity. Analysis of the *aa-nat* promoter revealed the presence of regulatory elements other than CREs that can potentially affect its transcription. For example, it contains an activating protein-1 (AP-1) site [38]. In the rat pineal gland, AP-1 homo- or heterodimeric transcription factors can be composed of immediate early genes *c-fos*, *c-jun*, *junB*, *junD*, *NGFI-A*, and *Fra-2* [39] and can display a daily variation in its binding activity with higher values during the night. This rhythmicity results from the accumulation of Fra-2 and JunB in the pineal gland during the nocturnal phase [40,41], and both factors function as transcriptional repressors [42,43]. STAT3 has been shown to stimulate transcription of *JunB* and *c-Fos* [44,45], and various modes of cooperation between STAT3 and other AP-1 factors have been reported [46,47,48]. If the basal level of STAT3 in the pineal gland maintains the basal levels of repressors, such as JunB and Fra-2, its reduction by STAT3 silencing may release a partial inhibition of night-time AANAT induction. 

Earlier studies have shown that, besides *aa-nat*, the NA/cAMP signaling pathway also induces the synthesis of inhibitory transcription factor ICER [49]. ICER inhibits the cAMP-stimulated gene expression by competing with CREB for the CRE consensus site. *Aa-nat* gene expression is increased following the *Icer* gene silencing either in vivo [50] or in vitro [32]. STAT3 could affect this ICER-driven inhibition directly, as the consensus STAT3 binding site exists on the *Icer* promoter that has been shown responsible for BDNF-induced *Icer* transcription in primary hippocampal neurons [51]. We measured the transcriptional level of *Icer* in the primary cultures treated with *Stat3* siRNA. Although ISO enhanced the *Icer* mRNA level as expected [52], the *Stat3* silencing did not show any effect. We cannot conclude that the STAT3 action is specific to AANAT, but we may suggest that the regulatory role of STAT3 is not universal to all ISO-induced genes in the pineal gland. Furthermore, in cultured pinealocytes and in contrast to the hippocampus [51], the expression of *Icer* does not seem to be dependent on STAT3. 

The promoter of the *Stat3* gene also contains its own consensus binding site [14], and thus its expression can be regulated by the positive autoregulatory loop. Our data show that siRNA significantly downregulates the *Stat3* mRNA in both control and ISO-stimulated pinealocytes, which proves the autoregulatory loop of *Stat3* transcription in pineal glands, as well as the efficiency of our experimental setup.

The fundamental property of the pineal gland’s melatonin synthetic pathway is its sensitivity to nocturnal light, which rapidly downregulates the *aa-nat* transcription and AANAT activity and, thus, decreases the melatonin level within minutes. As the STAT3 silencing clearly enhances AANAT activity, we tested the hypothesis that the STAT3 inhibition could interfere with light-induced AANAT suppression and rescue, at least partly, the AANAT activity. We have used a 1-min light pulse of intensity 30 lux applied in the late subjective night and showed that the light pulse reliably suppressed the AANAT activity within 20 min. However, the STAT3 silencing had no effect. The AANAT activity from the pineal glands of animals treated with STAT3 inhibitors was suppressed to the same extent as in untreated animals. It is possible that the combination of pulse duration and intensity exceeded the limit for saturation in nocturnal rodents, which the modulatory effects of STAT3 inhibition could not surpass. The 5-h interval between acute administration of inhibitors and the light pulse or tissue harvest has been designed based on in vitro studies [23]. Since then, these inhibitors were used in long-term chronic experiments [53] or shorter intervals between co-stimulatory agents [54]. Although a 5-h interval is sufficient to obtain a significant change in basal AANAT activity, it may not be optimal for subsequent stimulation with the light pulse. It is possible that more time- and dose-focused experiments could provide more precise data.

Our recent work has focused on the LPS-induced changes in the rat circadian system [18,19]. Within those studies, we have revealed that systemic LPS administration rapidly induces levels of pSTAT(y) and pSTAT3(s) in the SCN. Many other laboratories have repeatedly shown that LPS and pro-inflammatory cytokines induce STAT3 phosphorylation in a variety of tissues in vivo, as well as in vitro [55,56,57]. Our present results show that, in the pineal gland, the LPS significantly enhances *Stat3* transcription for more than 13 h and pSTAT3 levels for longer than 15 h. Number of pSTAT(y) immunopositive cells was slightly, but significantly, higher even 26 h from the LPS administration. In the SCN, the level of LPS-induced pSTAT3 was at its maximum 5 h after injection, while in the pineal gland, the maximum induction had occurred by the first-time measurement, 2 h from the LPS administration. The difference is probably the consequence of faster exposure to the blood circulating LPS in the pineal gland as a circumventricular organ.

Some reports focused on the effect of LPS and pro-inflammatory cytokines on AANAT and melatonin levels in the pineal gland demonstrated that LPS, TNF-α, or IL-1𝛽 supress the *aa-nat* transcription [33,58,59]. As the LPS upregulates the STAT3 in the pineal gland and the STAT3 dampens the *aa-nat* transcription and activity, we hypothesized that LPS-induced STAT3 upregulation can play a role in LPS-induced AANAT downregulation. We performed a set of in vivo and in vitro experiments, but our results did not prove the LPS-induced downregulation of *aa-nat* transcription in vitro and AANAT activity in vivo. LPS alone did not affect *Icer* transcription but enhanced *Stat3* mRNA in cultured pinealocytes, similarly to in vivo, which has proved the effectiveness of our LPS cell treatments. 

The principal studies [33,59] have demonstrated that LPS induces TNF-α in cultured pineal glands within 2–4 h and that TNF-α inhibited NA-induced *aa-nat* transcription within the interval 0.5–12 h from the administration. Although our experimental setup is in time-course with this study, it is possible that methodical differences, such as culturing conditions or dosing, could differ from our study. In support, a recent study shows that IFN-γ enhances the *aa-nat* mRNA in the dose 100 ng/mL, but the dose 30 ng/mL has no effect [60]. Furthermore, in ewes, the direction of the response of pineal AANAT to intracerebroventricular injection of IL-1𝛽 depends on the photoperiod [61], suggesting more complex interactions between the immune system and the melatonin synthetic pathway. Interestingly, the ISO-induced *Icer* transcription reflected the presence of LPS more significantly than the *aa-nat* did, as was similarly observed in macrophages [62].

Our data show that STAT3 inhibition has a potentiating effect on the ISO-induced *aa-nat* mRNA level, as well as on night/ISO-induced AANAT activity. These results could implicate that LPS-enhanced STAT3 will lead to the more effective suppression of AANAT, which, however is not the case. The immune-related changes in *aa-nat* expression and the melatonin synthetic pathway have been recently attributed to the STAT1–NFκB crosstalk [60]. It is possible that STAT3 participates only in homeostatic day/night AANAT regulation, and its LPS-induced pool plays a different role in pineal gland physiology, for example upregulation of serine protease inhibitors release as suggested earlier [63]. Alternatively, the STAT3 can play a role other than as a transcription factor. In the hippocampus, the STAT3 plays an essential role in the induction of NMDA-receptor-dependent long-term depression independently from transcription activity, suggesting its involvement in synaptic plasticity [64]. It activates various microRNAs, which broadens its role to many physiological processes in the cell, including molecular clockwork [65,66]. Extensive data from the literature suggest that the JAK-STAT3 pathway is seriously considered a potential therapeutic target for cancer treatment [67,68,69]. Therefore, additional research is essential to better understand the basal, non-pathological significance of STAT3 for physiology, including the circadian system and melatonin production.

## Data Availability

Data are contained within the article or supplementary material.

## Supplementary Materials

The following are available online at https://www.mdpi.com/article/10.3390/life11101105/s1, Figure S1: The western blots of STAT3 and its phosphorylated forms after transfection of pinealocytes with *Stat3* siRNA, Figure S2: The western blot of TLR4 receptor in three randomly selected pinealocytes cultures.
