# Supplementary material for: The Circadian Rhythms of STAT3 in the Rat Pineal Gland and Its Involvement in Arylalkylamine-N-Acetyltransferase Regulation
Source: Life (Basel). 2021 Oct 18;11(10):1105. doi: 10.3390/life11101105 (PMC8541109; doi:10.3390/life11101105)
Supplement: Supplementary file 1 [file life-11-01105-s001.zip › Supplementary Materials.pdf]

Supplementary Information for: The Circadian Rhythms of STAT3 in the Rat Pineal Gland and Its Involvement in Arylalkylamine-N-acetyltransferase Regulation

Simona Moravcová, Eva Filipovská, Veronika Spišská, Irena Svobodová, Jiří Novotný, Zdeňka Bendová*

***Zdeňka Bendová (zdenka.bendova@natur.cuni.cz)**

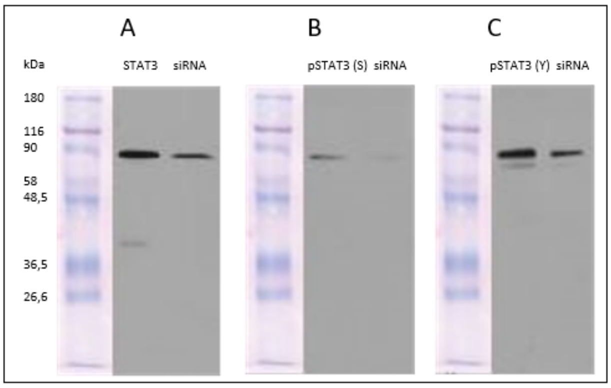

Figure S1. The original western blots of STAT3 (**A**) and its phosphorylated forms (**B**, **C**) after transfection of pinealocytes with *Stat3* siRNA.

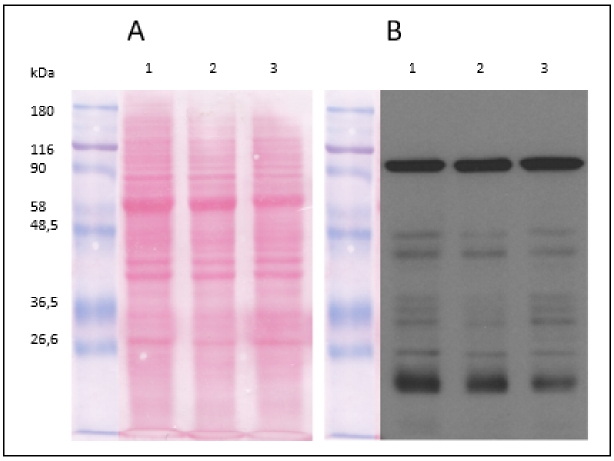

Figure S2. The original western blot of TLR4 receptor in three randomly selected pinealocytes cultures. Protein transferred to nitrocellulose membrane were first stained with Ponceau S (**A**) and then TLR4 was detected with specific antibodies (**B**).
